# Unmet Needs in Treatment Escalation for Chronic Spontaneous Urticaria: Findings From the CURE Registry

**DOI:** 10.1111/all.70199

**Published:** 2026-01-14

**Authors:** Pavel Kolkhir, Pascale Salameh, Magdalena Zajac, Alicja Kasperska‐Zajac, Ana Giménez‐Arnau, Maria Puertolas, Hanna Bonnekoh, Carolina Vera Ayala, Michael Makris, Eleni Chatzidimitriou, Stamatios Gregoriou, Kanokvalai Kulthanan, Andrea Bauer, Mojca Bizjak‐Suran, Daria Fomina, Alexis Bocquet, Joachim Dissemond, Mohamed Abuzakouk, Tara Raftery, Nadine Chapman‐Rothe, Emek Kocatürk, Clive Grattan, Riccardo Asero, Jonny G. Peter, Simon Francis Thomsen, Karsten Weller

**Affiliations:** ^1^ Institute of Allergology, Charité – Universitätsmedizin Berlin, Corporate Member of Freie Universität Berlin and Humboldt‐Universität Zu Berlin Berlin Germany; ^2^ Fraunhofer Institute for Translational Medicine and Pharmacology ITMP, Immunology and Allergology Berlin Germany; ^3^ School of Medicine Lebanese American University Byblos Lebanon; ^4^ Institut National de Santé Publique D'épidémiologie Clinique et de Toxicologie‐Liban (INSPECT‐LB) Beirut Lebanon; ^5^ Department of Primary Care and Population Health University of Nicosia Medical School Nicosia Cyprus; ^6^ Faculty of Pharmacy Lebanese University Hadat Lebanon; ^7^ European Center for Diagnosis and Treatment of Urticaria Medical University of Silesia Katowice Poland; ^8^ Department of Dermatology Hospital del Mar Research Institute, Universitat Pompeu Fabra Barcelona Spain; ^9^ Allergy Unit “D. Kalogeromitros”, 2nd Department, Dermatology and Venereology, National and Kapodistrian University of Athens, “Attikon” University Hospital Athens Greece; ^10^ National and Kapodistrian University of Athens, 1st Department of Dermatology‐Venereology, Andreas Sygros Hospital Athens Greece; ^11^ Department of Dermatology, Faculty of Medicine Siriraj Hospital Mahidol University Bangkok Thailand; ^12^ Department of Dermatology University Hospital Carl Gustav Carus, Technical University Dresden Dresden Germany; ^13^ Division of Allergy University Clinic of Respiratory and Allergic Diseases Golnik Golnik Slovenia; ^14^ Faculty of Medicine University of Ljubljana Ljubljana Slovenia; ^15^ Faculty of Maribor University of Maribor Maribor Slovenia; ^16^ Moscow City Research and Practical Center of Allergy and Immunology, State Budgetary Healthcare Institution “Moscow Clinical Science and Research Hospital 52 of Moscow Healthcare Department” Moscow Russian Federation; ^17^ Department of Clinical Immunology and Allergy, Sechenov First Moscow State Medical University Moscow Russian Federation; ^18^ Department of Pulmonology Astana Medical University Astana Kazakhstan; ^19^ Department of Internal Medicine and Clinical Immunology CHU Grenobles Alpes Grenoble France; ^20^ Department of Dermatology, Venerology and Allergology University of Essen Essen Germany; ^21^ Allergy & Immunology Department Cleveland Clinic Abu Dhabi Abu Dhabi UAE; ^22^ Novartis Ireland Ltd. Dublin Ireland; ^23^ Novartis Pharma AG Basel Switzerland; ^24^ Bahçeşehir University School of Medicine, Department of Dermatology Istanbul Turkey; ^25^ Guy's Hospital, St John's Institute of Dermatology London UK; ^26^ Ambulatorio di Allergologia, Clinica San Carlo, Paderno Dugnano Milan Italy; ^27^ Allergy and Immunology Unit, UCT Lung Institute Cape Town South Africa; ^28^ Division of Allergy and Clinical Immunology, Groote Schuur Hospital University of Cape Town (UCT) Cape Town South Africa; ^29^ Department of Dermatology Bispebjerg Hospital Copenhagen Denmark; ^30^ Department of Biomedical Sciences University of Copenhagen Copenhagen Denmark

**Keywords:** antihistamine, chronic spontaneous urticaria, chronic urticaria registry (CURE), real‐world practice, treatment escalation

## Abstract

**Background:**

Many patients with chronic spontaneous urticaria (CSU) remain symptomatic despite receiving second‐generation H_1_‐antihistamines (sgH_1_‐AH). This data analysis from the Chronic Urticaria Registry (CURE) aimed to describe treatment patterns and identify unmet needs in real‐world practice.

**Methods:**

CURE is an international, prospective registry of patients with chronic urticaria. Treatment responses were categorized as Urticaria Control Test (UCT) changes from baseline (BL) to 6‐month follow‐up (FU). Complete response was defined as UCT = 16 with a ≥ 3‐point increase.

**Results:**

Data were available from 3995 adult patients with CSU at BL and 1288 at FU with evaluable UCT. After treatment escalation from BL to FU, 5.3% (no treatment to licensed‐dose sgH_1_‐AH), 6.0% (licensed‐dose sgH_1_‐AH to up‐dosed sgH_1_‐AH), and 28.4% (any dose sgH_1_‐AH to omalizumab) achieved complete response. Factors associated with a lower probability of treatment escalation at FU were UCT ≥ 12 and omalizumab treatment at BL (both *p* < 0.0001). About one‐third (28.6%) of patients clinically eligible for escalation at BL (UCT < 12) did not receive step‐up treatment (18.0%) or were even stepped down (10.6%) and remained poorly controlled at FU. Factors associated with lack of escalation in this group included younger age (*p* = 0.014), shorter disease duration (*p* = 0.071), presence of wheals and angioedema (*p* = 0.002), better quality of life (*p* = 0.001), and treatment with up‐dosed sgH_1_‐AH (*p* = 0.031).

**Conclusion:**

Appropriate treatment escalation improves CSU control, although only about a quarter of patients achieve a complete response, indicating the need for novel treatments. Many patients with poorly controlled CSU do not receive guideline‐recommended treatment escalation and remain symptomatic on their current treatments, which deserves further attention.

## Graphical abstract

Appropriate treatment escalation improves CSU disease control. However, only about a quarter of patients achieve a complete response, the main goal of CSU treatment. Approximately one‐third of patients clinically eligible for escalation (UCT < 12) do not receive guideline‐recommended treatment escalation and remain symptomatic on their current treatments. CSU, chronic spontaneous urticaria; DLQI, Dermatology Life Quality Index; *N*, total number of patients; sgH_1_‐AH second‐generation H_1_‐antihistamine; UCT, urticaria control test.

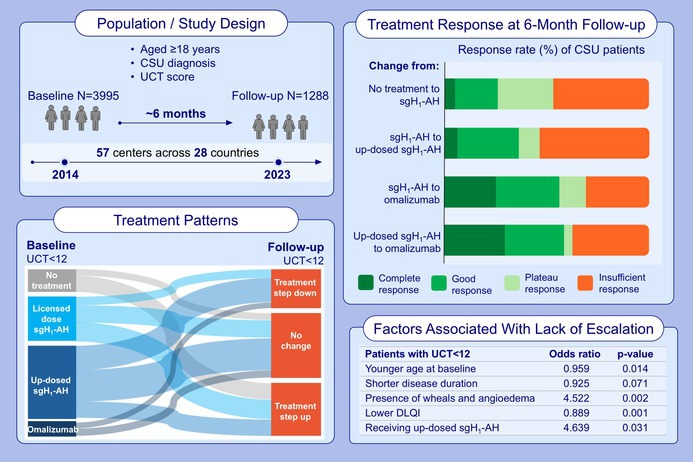

## 1 Introduction

Chronic spontaneous urticaria (CSU) presents as itchy wheals (hives), angioedema, or both for > 6 weeks and is estimated to affect approximately 0.5%–1.4% of the population globally [1, 2, 3]. Hives and angioedema develop spontaneously without specific or definite triggers for their occurrence [4]. CSU is a distressing disease that negatively impacts many aspects of patients' quality of life (QoL), including sleep, mental health, and performance, and may present with comorbidities [3, 5]. CSU is associated with an increased risk of mortality, likely due to comorbidities that are the leading causes of death, particularly suicide and cerebrovascular diseases [6]. Effective treatment and management of CSU symptoms may improve morbidity and mortality.

International urticaria guideline recommends second‐generation H_1_‐antihistamines (sgH_1_‐AH) as the first‐line treatment for CSU, including sgH_1_‐AH dose escalation up to 4 times the licensed dose [3, 4]. Omalizumab, a monoclonal anti‐IgE antibody, is recommended as the second‐line treatment for patients who do not show sufficient improvement with sgH_1_‐AH, at the guideline‐recommended dose for CSU (300 mg every 4 weeks). Patients who do not respond to standard omalizumab can be treated at higher doses, shorter intervals or both (off‐label practice). For patients who remain unresponsive to omalizumab treatment, ciclosporin, which is off‐label for urticaria, is recommended [4]. The disease is considered completely controlled (the main goal of CSU treatment) once a score of 16 is achieved on the Urticaria Control Test (UCT), a validated patient‐reported outcome measure (PROM) to assess disease control [7, 8]. Regardless of treatment, step‐down is recommended when the patient is symptom‐free for 3–6 months, based on individual patient factors and disease scenario, such as reducing the dose or extending intervals [4, 7, 9]. In case of relapse following treatment discontinuation or step‐down, patients should resume or step up treatment as soon as possible [7]. Consequently, stepping up and stepping down treatment is expected in CSU disease management [4, 10, 11].

The objective of this analysis was to describe real‐world treatment patterns and identify unmet needs, with particular focus on treatment escalation, the clinical outcome, and the response to treatment changes in patients with CSU prospectively collected through the chronic urticaria registry (CURE) from baseline (BL) to 6‐month follow‐up (FU). Notably, this study differs from previous reports such as AWARE which collected data from office‐based dermatologists and Urticaria Voices which was a patient‐based survey [12, 13]. In contrast, data collected through CURE were from specialized urticaria centers, mostly within the Global Allergy and Asthma European Network (GA^2^LEN) Urticaria Centers of Reference and Excellence (UCARE) network [14, 15].

## 2 Methods

### 2.1 Study Design

CURE is an investigator‐initiated, prospective, multicenter, observational, disease registry study that collects data in 79 centers globally [14, 16]. CURE is the first international disease registry that aimed to enhance our knowledge and understanding of chronic urticaria (CU) and urticarial vasculitis, including clinical course and outcomes, disease burden, treatment patterns and response, comorbidities and health care utilization [16]. Data for this analysis were collected from December 2015 to April 2023 through CURE using physician‐reported and/or patient‐reported questionnaires, as previously described [14, 16], to assess treatment patterns at BL and 6‐month FU visits in patients aged ≥ 18 years with a confirmed diagnosis of CSU. Data extracted for this analysis were collected from 28 countries across Europe, Asia, Africa, and Latin and South America, and most were GA^2^LEN UCARE centers [14, 15]. A list of participating centers is provided (Table S1). Anonymized data were entered into the registry by the treating physician or other medical staff.

### 2.2 Ethics

The CURE registry study was approved in 2014 and 2023 by the Ethics Committee of the Charité—Universitätsmedizin Berlin, Germany (reference EA1/146/14 and EA1/173/24). Before joining CURE, all participating centers obtained ethics approval from their relevant ethics committees. The participants provided written informed consent to have their data entered into CURE.

### 2.3 Outcome Measures

Assessments included demographics, clinical characteristics, and UCT score. Treatment patterns and outcomes were assessed at BL and FU in patients on no treatment or the guideline‐recommended therapies of licensed‐dose sgH_1_‐AH, up‐dosed sgH_1_‐AH (up to 4× licensed dose), omalizumab (sgH_1_‐AH permitted), ciclosporin (sgH_1_‐AH permitted), or “other treatment”. The “other treatment” category included any combination of the following medications: first‐generation antihistamines (AH) at standard or high dose (alone or in combination), non‐exclusive sgAH, systemic steroids, dapsone, methotrexate, autologous blood injections, and antibiotics, if it was the “current” treatment (in the previous 4 weeks) at the BL or FU visit.

Treatment patterns of patients over time were analyzed based on those receiving no treatment, licensed‐dose sgH_1_‐AH, up‐dosed sgH_1_‐AH, and omalizumab (licensed dose) from BL to FU. CSU was defined as completely controlled (UCT = 16), well‐controlled (UCT = 12–15), and poorly controlled (UCT < 12).

In this analysis, levels of disease control in response to treatment were assessed as UCT score changes from BL to FU. A 3‐point difference in the UCT score is considered to provide a clinically meaningful change for the patient [17]. Herein treatment response at FU was defined as follows: UCT = 16 with a ≥ 3‐point increase, complete response; UCT = 12–15 with a ≥ 3‐point increase, good response (well‐controlled CSU); UCT ≥ 12 with a < 3‐point increase, plateau response; and UCT < 12, insufficient response.

### 2.4 Statistical Analysis

The data collected were analyzed using Statistical Package for Social Sciences (SPSS) software, version 28.0 from IBM. Descriptive analysis involved reporting categorical variables as frequencies and percentages, and quantitative variables as means and standard deviations or medians and interquartile ranges (IQRs). Descriptive results of demographic, clinical, and management variables were shown for BL and FU. In addition, patients at BL were stratified according to their treatment categories and control level at BL, and the status at FU was described. The patients included in the omalizumab group may have been taking sgH_1_‐AH; however, for the purpose of this analysis, for patients who were prescribed omalizumab, it was considered their primary treatment and concomitant sgH_1_‐AH use was not analyzed.

For multivariable analysis, logistic regression analysis was performed because the dependent variables were dichotomous (treatment step‐up in general and specifically from sgH_1_‐AH to omalizumab). The Hosmer‐Lemeshow test was used to assess model adequacy. The selection of potential confounders was based on the clinical judgment of the investigators (not data‐driven), thereby reducing the risk of overfitting noise or missing important confounders [18]. This was followed by an ENTER model to be as comprehensive as possible, and the variables shown were the ones that had a significant association with the dependent variable. The ENTER method was used after including all variables of clinical interest, with beta coefficients and their exponential (adjusted odds ratio), 95% confidence intervals (CI), and *p*‐values reported. Sociodemographic and other relevant independent variables were added as appropriate, considering the allowed number of variables per model according to the sample size.

## 3 Results

### 3.1 Demographics and Disease Characteristics

Data were collected from 3995 adult patients with CSU, from 57 centers across 28 countries at BL. In the study population, 73% were female, the median (IQR) age was 44 (33–57) years, and the median (IQR) disease duration was 2.3 (0.8–6.8) years. The median (IQR) age at disease onset was 38.5 (27.8–51.7) years (Table 1). In total, 23.3% (880 of 3778) had concomitant chronic inducible urticaria (CIndU), of which the most frequent subtype was symptomatic dermographism (48.3%), followed by delayed pressure urticaria (35.6%) and cold urticaria (7.3%). UCT score was available for 3007 patients at BL, of whom 2501 were included in this analysis, while at FU, data were collected from 1288 patients, of whom 859 had UCT score available and 582 were included. This analysis focused on the treatment patterns of patients receiving no treatment, licensed‐dose sgH_1_‐AH, up‐dosed sgH_1_‐AH (up to 4× licensed dose), and omalizumab (licensed dose) (Figure S1). The median (IQR) interval between BL and FU visits was 6 (4.5–8.5) months. Sensitivity analysis demonstrated that patients with data available at FU generally had a slightly higher baseline disease activity (Urticaria Activity Score over a 7‐day period [UAS7], Dermatology Life Quality Index [DLQI], and Chronic Urticaria Quality of Life Questionnaire [CU‐Q_2_oL]) and were slightly older than those with no FU data (Table S2).

**TABLE 1:** Baseline demographics and disease characteristics of the patients.

Demographic/disease characteristic	Number
Participants, *N* (%)	3995 (100)
Asia	1492 (37.3)
Africa	118 (3.0)
Europe	2235 (55.9)
North America	3 (0.1)
South America	147 (3.7)
Age (years), median (IQR); *n*	44 (33–57); 3995
Female, % (*n*/*N*)	73.0 (2915/3995)
BMI (kg/m^2^), median (IQR); *n*	25.4 (22.4–29.4); 3387
Family history of CU, % (*n*/*N*)	8.4 (292/3480)
Presence of angioedema, % (*n*/*N*)	63.7 (2492/3915)
Disease duration in years, median (IQR); *n*	2.3 (0.8–6.8); 3879
Age at disease onset (years), median (IQR); *n*	38.5 (27.8–51.7); 3874
Diagnostic delay (days), median (IQR); *n*	31 (0–215); 3193
Concomitant CIndU^a^, % (*n*/total)	23.3 (880/3778)
UCT, median (IQR); *n*	8 (5–12); 3007
UAS7, median (IQR); *n*	15 (5–28); 1991
CU‐Q_2_oL, median (IQR); *n*	30 (15–45); 1474
DLQI, median (IQR); *n*	6 (2–13); 1833
Sleep disturbance, % (*n*/*N*)	45.2 (1490/3298)

Abbreviations: BMI, body mass index; CIndU, chronic inducible urticaria; CU, chronic urticaria; CU‐Q_2_oL; chronic urticaria quality of life questionnaire; DLQI, dermatology life quality index; IQR, interquartile range; *n*, number of patients; *N*, total number of patients; UAS7, weekly urticaria activity score; UCT, urticaria control test.

^a^ The most frequent subtypes of CIndU were symptomatic dermographism (48.3%), delayed pressure urticaria (35.6%), and cold urticaria (7.3%).

### 3.2 Appropriate Treatment Escalation Improves CSU Control, Although Many Patients Do Not Achieve Well‐Controlled or Completely Controlled Disease

**FIGURE 1:**
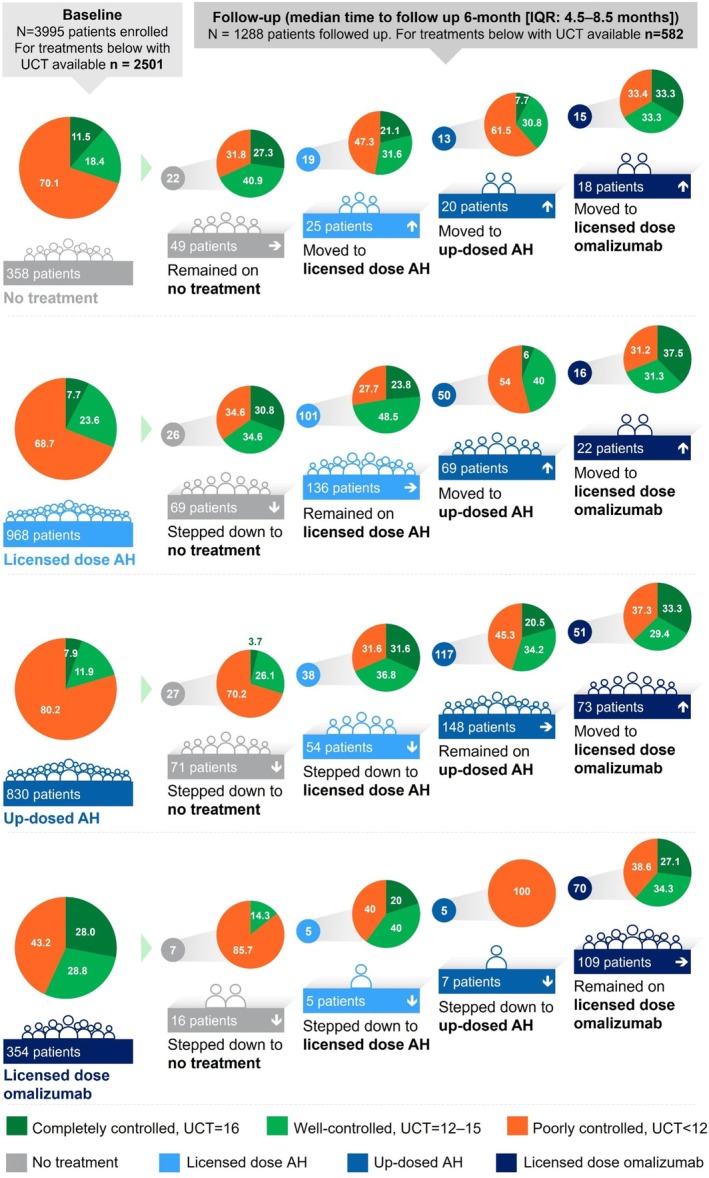
Treatment patterns from baseline to 6‐month follow‐up in patients with CSU. AH licensed dose: Only patients with monotherapy are included in this group. Up‐dosed AH: Dose not exceeding the 4× licensed dose can be a combination of H_1_‐AH. The ciclosporin group was not included due to the relatively small patient number receiving ciclosporin at baseline (0.6%, 24/3995 [%, *n*/total]). Treatment patterns were defined as follows: Completely controlled (UCT = 16), well‐controlled (UCT = 12–15), and poorly controlled (UCT < 12). Interpretation of data from Panel A – no treatment at BL, reading left to right: Panel (A) Of the patients on no treatment at baseline, 49 remained on no treatment (22 of whom had UCT available), 25 were escalated to licensed‐dose sgH_1_‐AH, 20 patients were escalated to up‐dosed sgH_1_‐AH, and 18 to omalizumab at FU. Of the 25 patients who escalated to licensed‐dose sgH_1_‐AH, 19 had a UCT available and 47.3% of them had UCT < 12. Of the 20 patients who escalated to up‐dosed sgH_1_‐AH, 13 had a UCT score available, of which > 60% had poorly controlled disease at FU. Of the 18 patients who escalated to licensed‐dose omalizumab, 15 had a UCT available, of whom 66.6% had completely controlled or well‐controlled disease. Panels (B–D) follow the same structure. As shown in Table 2, a proportion of patients were on high dose omalizumab (1.1% at BL; 1.8% at FU), ciclosporin (at BL = 0.6%; at FU = 1.4%), and other treatments (at BL = 13.7%; at FU = 13.7%), had UCT score available and were outside the focus of the main analysis. AH, antihistamine; CSU, chronic spontaneous urticaria; IQR, interquartile range; *n*, number of patients included in analysis; *N*, total number of patients; sgH_1_‐AH, second‐generation H_1_ antihistamines; UCT, urticaria control test.

Compared with BL, the use of licensed‐dose omalizumab had increased at 6‐month FU (20.7% vs. 11.4%) and that of sgH_1_‐AH had decreased (licensed‐dose: 19.9% vs. 32.5%, up‐dosed: 22.3% vs. 27.2%, any dose: 42.2% vs. 59.7%) in the study population (Table 2). The proportion of patients with UCT = 12–15 and UCT = 16 increased at FU, overall (Figure 1). Appropriate treatment escalation improved CSU control, although many patients did not achieve well‐ or completely controlled disease. Overall, 20.7% (34 of 164) and 55.5% (91 of 164) achieved complete control (UCT = 16) and well/complete control (UCT ≥ 12), respectively, with any treatment switch (Figure 1). Of the patients receiving no treatment at BL (Figure 1A), 49 remained on no treatment, 25 were escalated to licensed dose sgH_1_‐AH and 20 were escalated to up‐dosed sgH_1_‐AH at FU. Treatment escalation from no treatment to licensed‐dose sgH_1_‐AH, and from licensed‐dose sgH_1_‐AH to up‐dosed sgH_1_‐AH resulted in about half of the patients reaching UCT ≥ 12 at 6‐month FU (52.7% and 46.0%, respectively). Treatment escalation to omalizumab from no treatment, licensed‐dose sgH_1_‐AH and up‐dosed sgH_1_‐AH resulted in more than half of the patients reaching UCT ≥ 12 at 6‐month FU: 66.6%, 68.8%, and 62.7%, respectively (Figure 1A–C).

**TABLE 2:** Percentage of patients with CSU receiving current treatments at baseline and 6‐month follow‐up.

Treatment group	Treatment	Patients	
		Baseline % (*n*/*N*)	Follow‐up % (*n*/*N*)
1	No treatment	13.4 (536/3995)	20.3 (261/1288)
2	sgH_1_‐AH licensed dose	32.5 (1299/3995)	19.9 (256/1288)
3	sgH_1_‐AH up‐dosed	27.2 (1087/3995)	22.3 (287/1288)
4	Omalizumab licensed dose	11.4 (456/3995)	20.7 (267/1288)
5	Omalizumab high dose	1.1 (45/3995)	1.8 (23/1288)
6	Ciclosporin	0.6 (24/3995)	1.4 (18/1288)
7	Other treatment	13.7 (548/3995)	13.7 (176/1288)

*Note:* Group 1: No treatment; Group 2: sgH_1_‐AH licensed dose: only patients who used second‐generation sgH_1_‐AH as a monotherapy were included in this group (Once prescribed an additional treatment [e.g., steroids, H_2_‐AH, leukotriene antagonists, combination with first‐generation AH], they have been moved to “other” group or omalizumab or ciclosporin group); Group 3: sgH_1_‐AH up‐dosed: all patients receiving sgH_1_‐AH monotherapy at a higher than the licensed dose; Group 4: Omalizumab licensed dose: additional sgH_1_‐AH are possible; Group 5: Omalizumab high dose: patients who received a higher dose in 4‐weekly intervals as well as patients with a licensed‐dose but in shortened intervals (less than 4 weekly), additional sgH_1_‐AH are possible; Group 6: Ciclosporin (additional sgH_1_‐AH are possible in licensed dose or high dose); Group 7: Other treatment included any combination of the following medications: first‐generation AH at a standard or high dose (alone or in combination), non‐exclusive second‐generation AH, systemic steroids, dapsone, methotrexate, autologous blood injections, and antibiotics, if it is the “current” treatment at the baseline or 6‐month follow‐up visit.

Abbreviations: AH, antihistamines; CSU, chronic spontaneous urticaria; *n*, number of patients on specified treatment; *N*, total number of patients; sgH_1_‐AH, second‐generation H_1_‐antihistamines.

**FIGURE 2:**
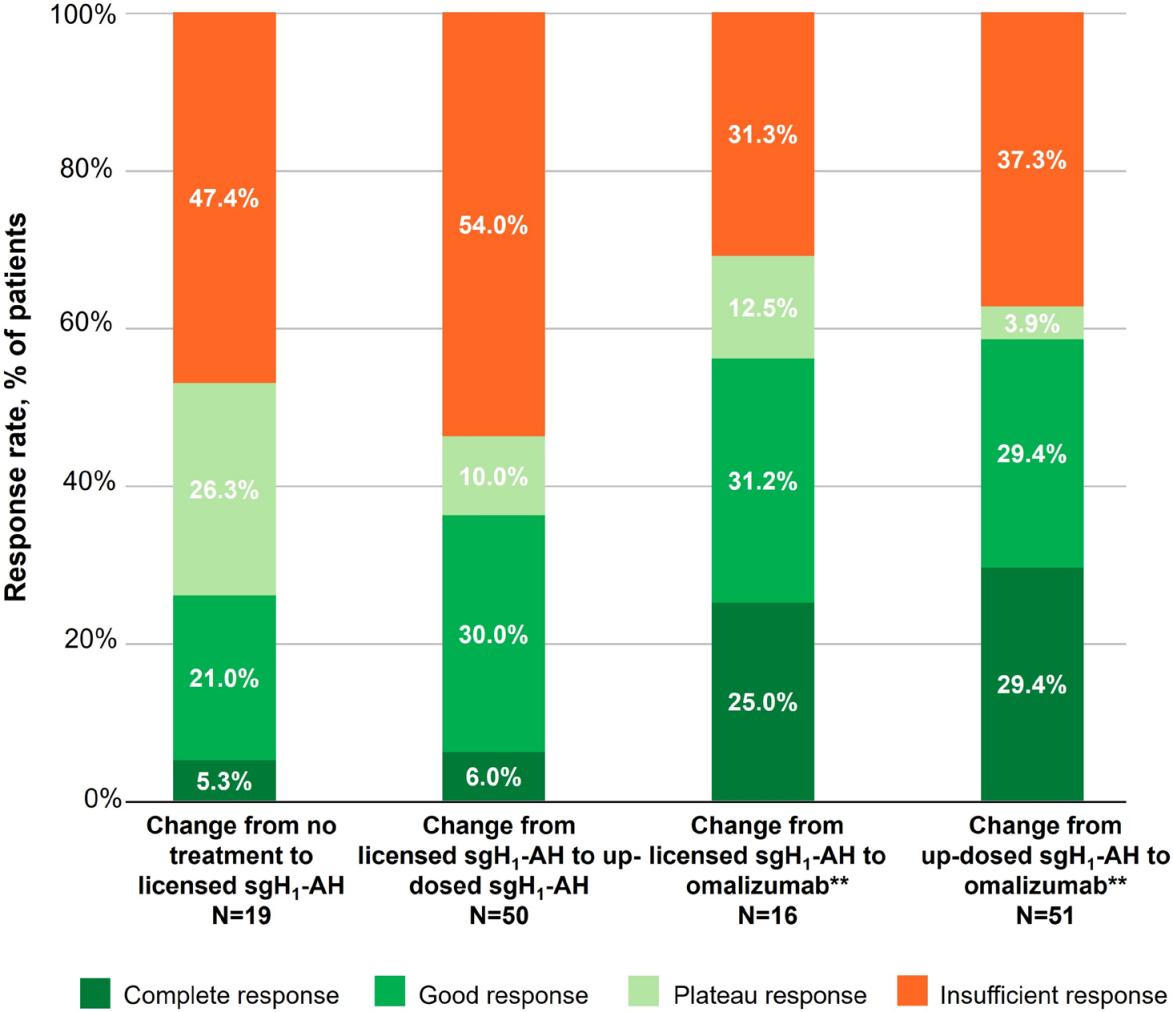
Treatment response based on UCT scores between baseline and 6‐month* follow‐up. *Median (IQR) interval between BL and FU was 6 (4.5–8.5) months; **Licensed‐dose omalizumab. Complete response: UCT = 16 at FU with a ≥ 3‐point increase; good response (well‐controlled CSU): UCT = 12–15 at FU with a ≥ 3‐point increase; plateau response: UCT ≥ 12 at FU with a < 3‐point increase; insufficient response: UCT < 12 at FU. BL, baseline; CSU, chronic spontaneous urticaria; FU, follow‐up; IQR, interquartile range; *N*, number of patients with complete data available for treatment and disease control at both baseline and follow‐up; sgH_1−_AH, second‐generation H_1_‐antihistamines; UCT, urticaria control test.

A small proportion of patients achieved good (UCT = 12–15 at FU with ≥ 3‐point increase) and complete (UCT = 16 at FU with ≥ 3‐point increase) response on sgH_1_‐AH: escalation from untreated to licensed‐dose sgH_1_‐AH: 21.0% and 5.3%, respectively; escalation from licensed‐dose sgH_1_‐AH to up‐dosed sgH_1_‐AH: 30.0% and 6.0%, respectively. A greater number of patients responded to escalation to licensed‐dose omalizumab: escalation from licensed‐dose sgH_1_‐AH: 31.2% and 25.0%, escalation from up‐dosed sgH_1_‐AH: 29.4% and 29.4%, escalation from any dose of sgH_1_‐AH: 29.9% and 28.4%, respectively (Figure 2).

### 3.3 Factors Associated With a Higher Probability of Treatment Escalation Include UCT < 12 and Not Receiving Omalizumab Treatment at BL

Logistic regression analysis indicated that UCT < 12 (poorly controlled disease) and not receiving omalizumab treatment at BL were predictors of patients being more likely to have their treatment stepped up at FU (both *p* < 0.0001). Patients who were older and those who had the disease for a longer duration were more likely to step up treatment; however, the results did not reach statistical significance (Table 3). The analysis also suggests that patients treated in European centers were more likely to have treatment escalation than those treated in Asia and other regions (*p* = 0.076).

**TABLE 3:** Multivariable logistic regression analysis showing predictors of step‐up^a^ treatment for patients with CSU and predictors of lack of treatment escalation for a subgroup of patients with UCT < 12.

Dependent variable	Odds ratio	*p* ^b^	95% CI
*Predictors of step‐up treatment*			
Age at baseline	1.014	0.084	0.998, 1.031
Disease duration	1.034	0.072	0.997, 1.073
Disease control at baseline (UCT ≥ 12 vs. UCT < 12)	0.316	< 0.0001	0.168, 0.596
Treatment center in Europe (vs. Asia/other)	1.809	0.076	0.940, 3.481
Omalizumab treatment at baseline vs. sgH_1_‐AH	0.088	< 0.0001	0.029, 0.269
*Predictors of lack of treatment escalation in the UCT < 12 group*			
Age at baseline	0.959	0.014	0.928, 0.991
Disease duration	0.925	0.071	0.850, 1.007
Presence of wheals and angioedema	4.522	0.002	1.767, 11.573
DLQI	0.889	0.001	0.828, 0.954
Up‐dosed sgH_1_‐AH	4.639	0.031	1.150, 18.714

Abbreviations: CI, confidence interval; CSU, chronic spontaneous urticaria; DLQI, dermatology life quality index; sgH_1_‐AH, second‐generation H_1_‐antihistamines; UCT, urticaria control test; vs., versus.

^a^ Step‐up indicates patients who had stepped up treatment at FU from BL treatment.

^b^ Two‐sided.

### 3.4 About One‐Third of Patients Eligible for Treatment Escalation at BL Did Not Receive It and Remained Poorly Controlled at FU

**FIGURE 3:**
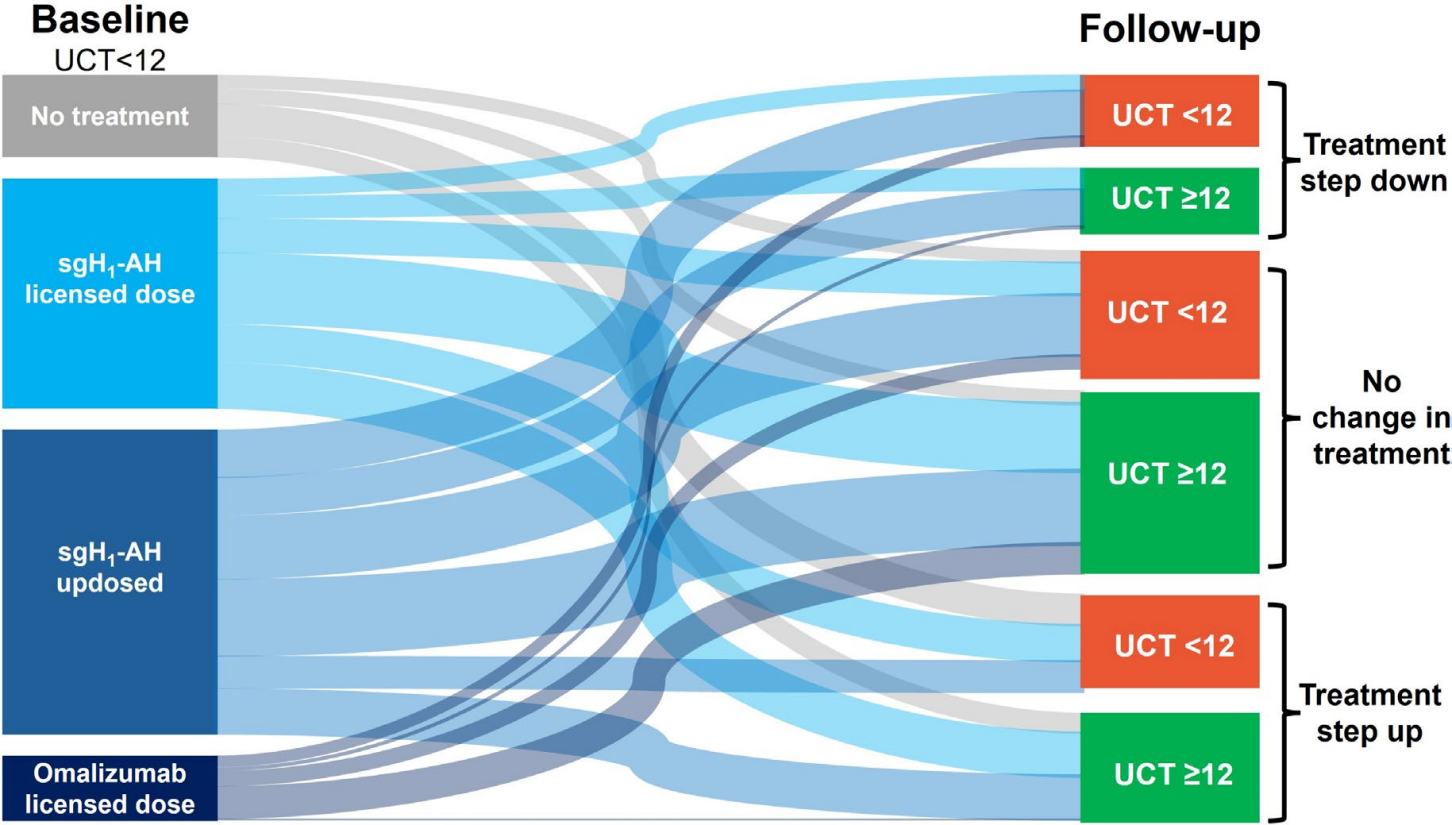
Treatment patterns in patients with CSU who had poorly controlled CSU (UCT < 12) at baseline. Sankey plot showing treatment patterns of patients with CSU who had a UCT < 12 at BL who had no change in treatment, stepped down treatment, or stepped up treatment and their level of disease control at FU. BL, baseline; CSU, chronic spontaneous urticaria; FU, follow‐up; sgH_1_‐AH, second‐generation H_1_ antihistamines; UCT, urticaria control test.

In total, 69.4% (293 of 422) patients with no treatment or receiving sgH_1_‐AH and who were eligible for escalation (UCT < 12 at BL) did not receive step‐up therapy (39.3%; 166 of 422) and were even stepped down (30.1%;127 of 422; Figure 3 and Table S3). In particular, in the no treatment, licensed‐dose sgH_1_‐AH and up‐dosed sgH_1_‐AH groups, 43.5% (*n* = 27 of 62), 40.5% (*n* = 62 of 153), and 37.2% (*n* = 77 of 207) of patients, respectively, eligible for escalation remained on BL treatments at FU despite having UCT < 12 at BL (Table S3).

Of the 322 patients with UCT available at FU, who had poorly controlled disease at BL (UCT < 12) and were receiving no treatment or sgH_1_‐AH, 45.3% (146 of 322) remained poorly controlled at FU (Table S3). About one‐third of the patients (28.6%, 92 of 322) clinically eligible for treatment escalation at BL remained on no treatment or sgH_1_‐AH (18.0%, 58 of 322) or were stepped down from sgH_1_‐AH (10.6%, 34 of 322) and remained poorly controlled at FU (Table S3).

### 3.5 Factors Associated With Lack of Treatment Escalation in the UCT < 12 Group of Patients at BL Include Younger Age, Shorter Disease Duration, Presence of Wheals and Angioedema, Better QoL, and Treatment With Up‐Dosed sgH_1_‐AH

Logistic regression analysis indicated that younger age (*p* = 0.014), shorter disease duration (*p* = 0.071), presence of wheals and angioedema (*p* = 0.002), better QoL (lower DLQI score, *p* = 0.001), and treatment with up‐dosed sgH_1_‐AH (*p* = 0.031) were associated with higher probability of not stepping up treatment for patients eligible for escalation at BL (UCT < 12; Table 3).

## 4 Discussion

The analysis of real‐world data of treatment patterns from CURE identified two unmet needs in CSU patient care. First, the frequency of patients who achieved complete control and well‐controlled urticaria generally increased over time and guideline‐recommended treatment escalation improved urticaria control. However, a considerable proportion of patients did not achieve complete control or well‐controlled disease despite stepping‐up treatment, pointing to the need for novel therapies. Second, many patients with poorly controlled CSU did not receive guideline‐recommended treatment escalation and remained symptomatic on their current treatment, which is a significant matter that requires further investigation.

Overall, about 50% of patients responded to treatment escalation and achieved successful alleviation of symptoms and improved disease control (UCT ≥ 12). However, despite available treatments, a significant proportion of patients failed to achieve well‐controlled or completely controlled disease. Few patients receiving no treatment at BL who were escalated to sgH_1_‐AH at FU achieved complete control, while about 50% did not respond sufficiently. Among those who received an increased dose of sgH_1_‐AH, the level of efficacy is in line with previous studies where the effectiveness of AH up‐dosing was variable [19, 20] and highlights the need for large, randomized studies to assess AH up‐dosing in CSU. A recent analysis of real‐world data from the United States (US) HealthVerity claims database, from January 2016–March 2023, showed that 59.1% of patients diagnosed with CSU had insufficiently controlled disease activity [21]. A more recent non‐interventional, cross‐sectional patient survey conducted in 2022 reported that 79% of patients with CSU were on AH, of which 84% had poorly controlled disease (UCT < 12). Of those taking AH, 53% were taking sgH_1_‐AH and 48% were taking first‐generation H_1_‐AH (of which 19% were taking both first‐generation AH and sgH_1_‐AH) [13]. Additionally, most patients (four of five) taking AH reported switching to another type of AH, and more than half reported up‐dosing of their current AH. Three of four patients who up‐dosed AH indicated either no relief (7%) or only partial improvement (68%) in their symptoms [13]. A systematic literature review reported that many patients with CSU had remained symptomatic despite treatment with standard doses of sgH_1_‐AH [22]. Some studies have reported improvement following escalation to higher doses AH in patients who did not respond to a standard dose, although limitations in the quality of these data were noted [22, 23].

Numerous clinical trials have concluded that omalizumab is efficacious in treating CSU, as defined by a reduction in urticaria activity and itch severity scores, and improved QoL, as reviewed by Casale et al. [24] In our study, escalation to omalizumab (300 mg dose) was effective in up to two‐thirds of patients, and high treatment persistence of omalizumab was observed. Patients receiving omalizumab who remained symptomatic may have been eligible for escalation to higher doses of omalizumab at the discretion of the treating physician. Patients who were receiving omalizumab at BL and those who had well‐controlled disease (UCT ≥ 12) were less likely to receive treatment escalation. This finding is consistent with our knowledge of the disease and recommended guidelines, as patients with well‐controlled disease, who are responding to treatment, do not require step‐up in treatment. Furthermore, the analysis revealed that patients who are older and those who have a longer disease duration are more likely to have dose escalation, suggesting a potential need to escalate treatment sooner in certain subgroups rather than allowing the disease to progress and negatively impact QoL for a prolonged period. The probability of patients being stepped up in treatment was similar across geographical regions, with patients from Europe having a higher likelihood of being stepped up, probably due to better availability of treatments such as omalizumab.

After treatment escalation from BL to FU, less than 10% of patients with CSU achieved complete response with any dose of sgH_1_‐AH and 28.4% with omalizumab. Previous analysis from CURE indicated similar rates, with 7% of patients treated with any dose of sgH_1_‐AH and 29% of those treated with omalizumab achieving complete control (UCT = 16) at BL [14]. Furthermore, we found that about one‐third of patients who were eligible for escalation were not stepped up at FU or were even stepped down despite not sufficiently responding to their current treatment. This finding is in line with observations from other real‐world studies [21, 25]. AWARE (A World‐wide Antihistamine‐Refractory chronic urticaria patient Evaluation) enrolled adult patients with CSU from urticaria centers and office‐based dermatologists and allergists across 12 European countries [25]. AWARE identified a high proportion of patients in Germany at enrollment who were receiving no treatment for CSU or were receiving treatment that was not guideline‐recommended, indicating undertreatment of patients at baseline [12, 25, 26]. Additionally, throughout the 1‐year observation period of AWARE in Germany, very low levels of treatment escalation were reported—up‐dosing from standard AH to up‐dosed AH—ranged from 0% to 6.7%, and escalation of treatment to omalizumab ranged from 0% to 11.1% [12].

Taking together, these results suggest the need to (1) identify and address physician‐ and patient‐related factors associated with the lack of treatment escalation, and (2) better understand difficult‐to‐treat CSU endotypes to develop more effective therapies. Physician‐related factors associated with the lack of treatment escalation can include low compliance with guidelines, limited access to treatment [27, 28], and poor physician education [25, 26]. The publication of international CU guidelines has established a standard of care for patients with CSU [4], contributing to improved disease management and a positive impact on patient's QoL [29]. However, adherence to the recommended guidelines in real‐world clinical practice is not consistent [29, 30]. Country‐specific factors influencing treatment patterns likely include local guidelines, financial constraints, and continuous medical education for healthcare providers. A global survey identified that while most physicians followed guidelines, more than 20% deviated from the guidelines while treating patients. Treating physicians who followed guidelines showed higher rates of using sgH_1_‐AH as first‐line therapy than those who did not follow guidelines [29]. Lack of adherence to the recommended guidelines is not unique to CSU disease management. Suboptimal medical care and disease management have been observed across other conditions globally, such as asthma and diabetes, implicating patients' QoL and disease severity [31, 32, 33, 34]. Guideline awareness and compliance can be increased with educational webinars, dedicated symposia and other activities [35, 36].

Patient‐related factors associated with lack of treatment escalation can include limited access to specialist healthcare, low patient awareness, communication and unwillingness to accept escalation to up‐dosed sgH_1_‐AH or addition of a monoclonal antibody. The results of the regression analysis showed that lack of step‐up in patients with poorly controlled CSU is associated with younger patient age, shorter disease duration, presence of wheals and angioedema, better QoL and treatment with up‐dosed sgH_1_‐AH. The latter points to the fact that some patients are reluctant to escalate to omalizumab. Shorter disease duration, younger age, and better QoL may explain that even if the disease is not well‐controlled, it is not burdensome, that is, without considerable QoL impairment, and it can still be tolerated by some (probably younger) patients. A possible explanation for the presence of both wheals and angioedema, which is usually a more severe CSU phenotype, in the UCT < 12 group of patients with CSU without escalation, might be the use of short course(s) of systemic corticosteroids that was not captured by this analysis.

The effectiveness of omalizumab as treatment for CSU in the real‐world setting has been highlighted across many studies [37], however barriers potentially exist to patients accessing omalizumab due to socioeconomic and healthcare system factors, particularly in low‐income regions. Financial constraints are considered a barrier to treatment adherence as reported across various geographical regions such as China, Hong Kong, and India [27, 28, 38, 39]. Conversely, in countries where access to specialist care and biologics, such as omalizumab, is not considered a barrier, such as the US and United Kingdom, disparity exists where socioeconomic circumstances, demographics, urban versus rural location, and health insurance policies are factors determining treatment adherence and management [40, 41, 42].

Poor response to available treatments can be linked to the underlying CSU mechanisms (endotypes), especially the autoimmune CSU endotype with mast cell‐activating IgG autoantibodies [43]. Novel treatments including disease‐modifying therapies that would help these and other subpopulations of patients with CSU are needed [44]. The treatment landscape for CSU will improve markedly during the next years, with new therapy options emerging [45, 46]. Dupilumab, a monoclonal antibody that blocks IL‐4Ra, significantly reduced urticaria activity in omalizumab‐naive patients and showed smaller effects in patients who were omalizumab‐intolerant/incomplete responders [46]. Remibrutinib, a Bruton's tyrosine kinase inhibitor, has shown efficacy in reducing disease symptoms in patients remaining symptomatic despite treatment with sgH_1_‐AH in clinical trials, without a notable drop in efficacy in biologic‐experienced patients, and it has recently been approved for use in adult patients with CSU by the US Food and Drug Administration [45, 47, 48]. However, this improved treatment landscape can only lead to better disease control if the current obstacles leading to lack of treatment escalation can be removed.

CURE is an observational registry that provides valuable insight into the real‐world experience of patients with CSU. However, there are limitations to the registry which are previously reported [14]. Although CURE is a global registry, most of the data available for analysis reported here were from Europe and Central Asia. There is a risk of selection bias because physicians are not obliged to register all patients consecutively. Particularly, for this analysis, patient data at FU were missing which resulted in fewer patients in some data sets; for example, there were only a small number of patients treated with ciclosporin at BL and FU, limiting the ability to draw meaningful conclusions for this group. Data for a large proportion of patients at FU may not have been available for a variety of reasons, such as a patient experiencing a less severe phenotype not returning for FU, spontaneous remission/intermittent remission, or patients waiting for the 6‐month appointment as the study is ongoing. Patients can stop attending physicians even when disease control has not been achieved [49]. Sensitivity analysis demonstrated that patients with data available at FU generally had a slightly higher baseline disease activity (UAS7, DLQI, and CU‐Q2oL), were also slightly older, and used significantly less standard‐dose sgH_1_‐AH and slightly more up‐dosed sgH_1_‐AH at BL than those not returning for the FU visit. Information about treatment at FU was recorded for the last 4 weeks, leaving a possibility that some patients might receive some other therapies between the BL and FU visits, with possible UCT score fluctuation. Although a multivariable analysis was conducted to reduce confounding, residual confounding due to unmeasured confounders cannot be ruled out.

In conclusion, our data from CURE revealed that many patients required treatment escalation from BL to FU. Among patients who stepped up their treatment with sgH_1_‐AH, at least half did not respond sufficiently. Escalation to omalizumab was effective in up to two‐thirds of patients with a high rate of treatment persistence. However, a complete response to treatment escalation (UCT = 16) was experienced by only about a quarter of patients, indicating the need for novel treatments. The lack of guideline‐recommended treatment escalation in patients with poorly controlled CSU highlights an important gap between evidence‐based recommendations and actual clinical practice—a point that deserves further investigation.

## Author Contributions

K.W. and P.K. contributed to the conceptualization, methodology, formal analysis, validation, visualization, and writing of the original draft. P.S. and T.R. contributed to the methodology, formal analysis, validation, visualization, and writing of the original draft. N.C.‐R. contributed to the formal analysis, validation, visualization, and writing of the original draft. All authors contributed to the drafting, reviewing, editing, and final approval of the manuscript.

## Funding

This study and work presented here were funded by Novartis Pharma AG, Basel, Switzerland.

## Conflicts of Interest

P. Kolkhir reports grants or contracts from Novartis Pharma AG and Sanofi; consulting fees from BioCryst, Insignia, Merus and ValenzaBio. P. Salameh, M. Zajac, A. Kasperska‐Zajac, M. Puertolas, C.V. Ayala, E. Chatzidimitriou, D. Fomina, A. Bocquet, M. Abuzakouk, R. Asero, S.F. Thomsen report no disclosures for the submitted work. A. Giménez‐Arnau reports grants or contracts from Escient Pharmaceuticals, Noucor, Novartis Pharma AG, Instituto Carlos III‐ FEDER, Uriach Pharma; consulting fees from Almirall, Amgen, Blue‐Print, Celldex, Escient Pharmaceuticals, FAES, Genentech, GlaxoSmithKline, Jaspers, Leo Pharma, Mitsubishi Tanabe, Novartis Pharma AG, Noucor, Sanofi–Regeneron, Thermo Fisher Scientific, Septerna, Servier, Uriach Pharma; payment or honoraria as a speaker for Almirall, Avene, Genentech, GlaxoSmithKline, LEO Pharma, Menarini, MSD, Noucor, Novartis Pharma AG, Sanofi, Uriach Pharma; support to attend scientific meetings from Almirall, Noucar; participation on an Advisory Borad for Celldex. H. Bonnekoh reports grants or contracts from Granular and Novartis Pharma AG; payment or honoraria as a speaker from AbbVie, Celltrion, Novartis Pharma AG, Sanofi Aventis, Sobi; participation as an advisor to Biocryst, ValenzaBio and Novartis Pharma AG. M. Makris reports payment or honoraria for presentations from Astra Zeneca, GlaxoSmithKline, Sanofi Aventis, Pfizer, Celldex, Elpen, Integris, Chiesi, Thermo Fisher, Menarini; travel support for attending scientific meetings from Chiesi, Sanofi Aventis and Menarini. S. Gregoriou reports payment or honoraria as a speaker, participation on an Advisory Board and travel support for attending scientific meetings from Sanofi, Novartis Pharma AG, AbbVie, Pfizer, Eli Lilly, LEO Pharma, Pierre Fabre and L'Oreal. K. Kulthanan reports payment or honoraria as a speaker from Novartis Pharma AG, Menarini and Sanofi Genzyme. A. Bauer reports research support from Sanofi, AbbVie, LEO Pharma, Novartis Pharma AG, Almirall; payment or honoraria as a speaker for Sanofi, AbbVie, LEO Pharma, Pfizer, Eli Lilly, Novartis Pharma AG, Behring, Almirall, Takeda; travel support from Sanofi, Takeda, BioCryst; participation as an advisor to KalVista Pharmaceuticals, Sanofi, LEO Pharma, Novartis Pharma AG and Behring; President for a working group for occupational and environmental dermatology. M. Bizjak reports payment or honoraria as a speaker from Novartis Pharma AG; participation on an Advisory Board for Novartis Pharma AG and Swixx BioPharma. J. Dissemond reports participation on an Advisory Board for Novartis Pharma AG. T. Raftery is an employee of Novartis Pharma AG Ireland Ltd., Dublin, Ireland and reports holding shares of Novartis Pharma AG. N. Chapman‐Rothe is an employee of Novartis Pharma AG, Basel, Switzerland, and reports holding shares of Novartis Pharma AG. E. Kocatürk reports grant or contract from Almirall; payment or honoraria as a speaker from Novartis Pharma AG and Menarini. C. Grattan reports consultancies for Celltrion, ThermoFisher and receiving sponsorship to attend a scientific meeting from Novartis Pharma. J.G. Peter reports grants from Takeda, KalVista Pharmaceuticals, Astria; consulting fees from Pharvaris, Takeda, Glanmark, Sanofi, Viatris, AbbVie; speaker fees from CSL Behring, Takeda; support to attend scientific meetings from Sanofi and HAE International; participation on an Advisory Board for Pharvaris and Astria. K. Weller reports medical writing support for this manuscript from Novartis Pharma AG; funding for CURE from Novartis Pharma AG, Sanofi, Noucor; participation on an Advisory Board for Novartis Pharma AG.

## Supporting information


**Table S1:** Participating CURE centers contributing to this analysis.
**Table S2:** Sensitivity analysis based on a comparison of baseline age, disease duration, disease activity scores, and use of sgH_1_‐AH between patients who had data at follow‐up versus those who did not have these data.
**Table S3:** Treatment patterns based on UCT score at baseline and follow‐up.
**Figure S1:** Data analysis flow chart. Analysis performed on data from patients receiving no treatment, licensed dose sgH_1_‐AH, up‐dosed sgH_1_‐AH (up to 4 times licensed dose) and omalizumab (licensed dose) with UCT score available at baseline and follow‐up.

## Data Availability

The data supporting the findings of this study are available from the corresponding authors upon request.

## References

[1] M. Maurer , K. Weller , C. Bindslev‐Jensen , et al., “Unmet Clinical Needs in Chronic Spontaneous Urticaria. A GA^2^LEN Task Force Report,” Allergy 66, no. 3 (2011): 317–330. 21083565 10.1111/j.1398-9995.2010.02496.x

[2] J. Fricke , G. Ávila , T. Keller , et al., “Prevalence of Chronic Urticaria in Children and Adults Across the Globe: Systematic Review With Meta‐Analysis,” Allergy 75, no. 2 (2020): 423–432. 31494963 10.1111/all.14037

[3] P. Kolkhir , H. Bonnekoh , M. Metz , and M. Maurer , “Chronic Spontaneous Urticaria: A Review,” JAMA 332, no. 17 (2024): 1464–1477. 39325444 10.1001/jama.2024.15568

[4] T. Zuberbier , A. H. Abdul Latiff , M. Abuzakouk , et al., “The International EAACI/GA^2^LEN/EuroGuiDerm/APAAACI Guideline for the Definition, Classification, Diagnosis, and Management of Urticaria,” Allergy 77, no. 3 (2022): 734–766. 34536239 10.1111/all.15090

[5] M. Sánchez‐Borges , I. J. Ansotegui , I. Baiardini , et al., “The Challenges of Chronic Urticaria Part 1: Epidemiology, Immunopathogenesis, Comorbidities, Quality of Life, and Management,” World Allergy Organization Journal 14, no. 6 (2021): 100533. 34221215 10.1016/j.waojou.2021.100533 PMC8233382

[6] P. Kolkhir , K. Bieber , T. Hawro , et al., “Mortality in Adult Patients With Chronic Spontaneous Urticaria: A Real‐World Cohort Study,” Journal of Allergy and Clinical Immunology 45 (2024): 1456. 10.1016/j.jaci.2024.11.036 39675681

[7] D. Terhorst‐Molawi , L. Fox , F. Siebenhaar , M. Metz , and M. Maurer , “Stepping Down Treatment in Chronic Spontaneous Urticaria: What we Know and What we Don't Know,” American Journal of Clinical Dermatology 24, no. 3 (2023): 397–404. 36810982 10.1007/s40257-023-00761-z PMC10195701

[8] K. Weller , A. Groffik , M. K. Church , et al., “Development and Validation of the Urticaria Control Test: A Patient‐Reported Outcome Instrument for Assessing Urticaria Control,” Journal of Allergy and Clinical Immunology 133, no. 5 (2014): 1365–1372. 24522090 10.1016/j.jaci.2013.12.1076

[9] A. M. Giménez Arnau , A. Valero Santiago , J. Bartra Tomás , et al., “Therapeutic Strategy According to Differences in Response to Omalizumab in Patients With Chronic Spontaneous Urticaria,” Journal of Investigational Allergology & Clinical Immunology 29, no. 5 (2019): 338–348. 30222111 10.18176/jiaci.0323

[10] J. Ceravalls , A. M. Giménez‐Arnau , V. Expósito‐Serrano , et al., “Redefining Omalizumab Discontinuation in Chronic Spontaneous Urticaria: The Value of Optimization and Predictive Factors of Relapse. A 52‐Week Multicenter Study,” Actas Dermo‐Sifiliográficas 116 (2025): 675–683. 40024599 10.1016/j.ad.2024.08.014

[11] J. Ceravalls , A. M. Giménez‐Arnau , V. Expósito‐Serrano , et al., “Predictive Factors and Optimization of Omalizumab in Chronic Spontaneous Urticaria: A Multicenter Study of 257 Patients,” Journal of Investigational Allergology and Clinical Immunology 35 (2025): 0. 10.18176/jiaci.1053 39950575

[12] M. Maurer , U. Raap , P. Staubach , et al., “Antihistamine‐Resistant Chronic Spontaneous Urticaria: 1‐Year Data From the AWARE Study,” Clinical and Experimental Allergy 49, no. 5 (2019): 655–662. 30415478 10.1111/cea.13309

[13] K. Weller , T. Winders , J. McCarthy , et al., “Urticaria Voices: Real‐World Experience of Patients Living With Chronic Spontaneous Urticaria,” Dermatol. Ther. (Heidelb.) 15 (2025): 747–761. 40019716 10.1007/s13555-025-01348-8 PMC11909366

[14] P. Kolkhir , P. A. Laires , P. Salameh , et al., “The Benefit of Complete Response to Treatment in Patients With Chronic Spontaneous Urticaria‐CURE Results,” Journal of Allergy and Clinical Immunology: In Practice 11, no. 2 (2023): 610–620. 36481420 10.1016/j.jaip.2022.11.016

[15] M. Maurer , M. Metz , C. Bindslev‐Jensen , et al., “Definition, Aims, and Implementation of GA(2) LEN Urticaria Centers of Reference and Excellence,” Allergy 71, no. 8 (2016): 1210–1218. 27038243 10.1111/all.12901

[16] K. Weller , A. Giménez‐Arnau , C. Grattan , et al., “The Chronic Urticaria Registry: Rationale, Methods and Initial Implementation,” Journal of the European Academy of Dermatology and Venereology 35, no. 3 (2021): 721–729. 32946615 10.1111/jdv.16947

[17] T. Ohanyan , N. Schoepke , B. Bolukbasi , et al., “Responsiveness and Minimal Important Difference of the Urticaria Control Test,” Journal of Allergy and Clinical Immunology 140, no. 6 (2017): 1710–1713. 28625805 10.1016/j.jaci.2017.04.050

[18] M. Z. I. Chowdhury and T. C. Turin , “Variable Selection Strategies and Its Importance in Clinical Prediction Modelling,” Family Medicine and Community Health 8, no. 1 (2020): e000262. 32148735 10.1136/fmch-2019-000262 PMC7032893

[19] M. Ferrer , J. Sastre , I. Jáuregui , et al., “Effect of Antihistamine Up‐Dosing in Chronic Urticaria,” Journal of Investigational Allergology and Clinical Immunology 21, no. Suppl 3 (2011): 34–39. 22185048

[20] K. Weller , A. M. Gimenez‐Arnau , J. Baron , et al., “Efficacy and Safety of On‐Demand Versus Daily Rupatadine in Chronic Spontaneous Urticaria: A Randomized Trial,” Allergy 79, no. 1 (2024): 93–103. 37597162 10.1111/all.15854

[21] M. A. Riedl , D. Patil , J. Rodrigues , et al., “Clinical Burden, Treatment, and Disease Control in Patients With Chronic Spontaneous Urticaria: Real‐World Evidence,” Annals of Allergy, Asthma & Immunology 25 (2024): 458. 10.1016/j.anai.2024.12.008 39694087

[22] S. Guillén‐Aguinaga , I. Jáuregui Presa , E. Aguinaga‐Ontoso , F. Guillén‐Grima , and M. Ferrer , “Updosing Nonsedating Antihistamines in Patients With Chronic Spontaneous Urticaria: A Systematic Review and Meta‐Analysis,” British Journal of Dermatology 175, no. 6 (2016): 1153–1165. 27237730 10.1111/bjd.14768

[23] P. Iriarte Sotés , M. Armisén , T. Usero‐Bárcena , et al., “Efficacy and Safety of up‐Dosing Antihistamines in Chronic Spontaneous Urticaria: A Systematic Review of the Literature,” Journal of Investigational Allergology and Clinical Immunology 31, no. 4 (2021): 282–291. 33030434 10.18176/jiaci.0649

[24] T. B. Casale , A. M. Gimenez‐Arnau , J. A. Bernstein , M. Holden , T. Zuberbier , and M. Maurer , “Omalizumab for Patients With Chronic Spontaneous Urticaria: A Narrative Review of Current Status,” Dermatol. Ther. (Heidelb.) 13, no. 11 (2023): 2573–2588. 37776480 10.1007/s13555-023-01040-9 PMC10613187

[25] M. Maurer , A. Giménez‐Arnau , L. F. Ensina , C. Y. Chu , X. Jaumont , and P. Tassinari , “Chronic Urticaria Treatment Patterns and Changes in Quality of Life: AWARE Study 2‐Year Results,” World Allergy Organization Journal 13, no. 9 (2020): 100460. 32983330 10.1016/j.waojou.2020.100460 PMC7493083

[26] M. Maurer , C. Costa , A. Gimenez Arnau , et al., “Antihistamine‐Resistant Chronic Spontaneous Urticaria Remains Undertreated: 2‐Year Data From the AWARE Study,” Clinical and Experimental Allergy 50, no. 10 (2020): 1166–1175. 32735720 10.1111/cea.13716

[27] H. W. F. Mak , V. Chiang , E. T. S. Chan , et al., “Disparities in Chronic Spontaneous Urticaria: Eligibility for Drug Reimbursement Associated With Clinical Outcomes,” Journal of Allergy and Clinical Immunology: Global 3, no. 2 (2024): 100243. 38585447 10.1016/j.jacig.2024.100243 PMC10997903

[28] H. W. F. Mak , F. K. L. Chung , P. H. Li , and M. Maurer , “Global Productivity, International Collaborations, and Research Trends in Chronic Spontaneous Urticaria: A Bibliometric Overview,” Journal of Allergy and Clinical Immunology: Global 4, no. 2 (2025): 100455. 40231228 10.1016/j.jacig.2025.100455 PMC11994327

[29] P. Kolkhir , D. Pogorelov , R. Darlenski , et al., “Management of Chronic Spontaneous Urticaria: A Worldwide Perspective,” World Allergy Organization Journal 11, no. 1 (2018): 14. 29988758 10.1186/s40413-018-0193-4 PMC6030778

[30] K. Weller , K. Viehmann , M. Bräutigam , et al., “Management of Chronic Spontaneous Urticaria in Real Life–In Accordance With the Guidelines? A Cross‐Sectional Physician‐Based Survey Study,” Journal of the European Academy of Dermatology and Venereology 27, no. 1 (2013): 43–50. 22150693 10.1111/j.1468-3083.2011.04370.x

[31] W. W. Busse and M. Kraft , “Current Unmet Needs and Potential Solutions to Uncontrolled Asthma,” European Respiratory Review 31, no. 163 (2022): 210176. 35082128 10.1183/16000617.0176-2021 PMC9488919

[32] K. M. Pantalone , A. D. Misra‐Hebert , T. M. Hobbs , et al., “Clinical Inertia in Type 2 Diabetes Management: Evidence From a Large, Real‐World Data Set,” Diabetes Care 41, no. 7 (2018): e113–e114. 29678811 10.2337/dc18-0116

[33] K. M. Pantalone , A. D. Misra‐Hebert , T. M. Hobbs , et al., “Intensification Patterns and the Probability of HbA(1c) Goal Attainment in Type 2 Diabetes Mellitus: Real‐World Evidence for the Concept of 'intensification Inertia',” Diabetic Medicine 37, no. 7 (2020): 1114–1124. 30653705 10.1111/dme.13900

[34] G. Reach , V. Pechtner , R. Gentilella , A. Corcos , and A. Ceriello , “Clinical Inertia and Its Impact on Treatment Intensification in People With Type 2 Diabetes Mellitus,” Diabetes & Metabolism 43, no. 6 (2017): 501–511. 28754263 10.1016/j.diabet.2017.06.003

[35] O. Yilmaz , I. Reisli , F. Tahan , F. Orhan , A. B. Boz , and H. Yuksel , “Influence of Education on Primary Care Physicians' Knowledge on Childhood Allergy as a Systemic Disease and the Atopic March,” Allergol Immunopathol (Madr) 39, no. 2 (2011): 73–78. 21208716 10.1016/j.aller.2010.03.012

[36] M. S. A. Calle , P. Schoonheim , M. Lebwohl , and A. Kaplan , “Online Education Yields Significant Gains in Physicians' Knowledge of Diagnosis and Management of Chronic Spontaneous Urticaria,” Journal of Allergy and Clinical Immunology 149, no. 2 (2022): AB177.

[37] J. A. Bernstein , A. Kavati , M. D. Tharp , et al., “Effectiveness of Omalizumab in Adolescent and Adult Patients With Chronic Idiopathic/Spontaneous Urticaria: A Systematic Review of 'real‐World' Evidence,” Expert Opinion on Biological Therapy 18, no. 4 (2018): 425–448. 29431518 10.1080/14712598.2018.1438406

[38] Y. Huang , H. Yuan , and Z. Huang , “Cost‐Utility Analysis of Omalizumab for the Treatment of Chronic Spontaneous Urticaria in China,” Cost Effectiveness and Resource Allocation 23, no. 1 (2025): 37. 40696383 10.1186/s12962-025-00643-7 PMC12285021

[39] M. Tiongco‐Recto , K. Woo , W. H. Chung , et al., “Prioritising Patient‐Centred Care in the Management of Chronic Urticaria in Asia‐Pacific Countries,” World Allergy Organization Journal 17, no. 11 (2024): 100984. 39553289 10.1016/j.waojou.2024.100984 PMC11564018

[40] M. Erlewyn‐Lajeunesse , L. P. Villa , S. Shaikh , et al., “Inequalities in Access to Specialist Allergy Services in the United Kingdom: A Report From the BSACI Registry for Immunotherapy (BRIT),” Clinical and Experimental Allergy 55, no. 6 (2025): 458–468. 40169378 10.1111/cea.70034 PMC12127059

[41] G. S. Mosnaim , M. Greenhawt , P. Imas , et al., “Do Regional Geography and Race Influence Management of Chronic Spontaneous Urticaria?,” Journal of Allergy and Clinical Immunology 150, no. 6 (2022): 1260–1264. 36481046 10.1016/j.jaci.2022.10.017

[42] S. Cheraghlou , N. Ugwu , B. Yu , and J. M. Cohen , “Trends in the Cost and Utilization of Omalizumab in the Medicare Population: 2013–2017,” Yale Journal of Biology and Medicine 95, no. 2 (2022): 207–212. 35782473 PMC9235260

[43] P. Kolkhir , M. Muñoz , R. Asero , et al., “Autoimmune Chronic Spontaneous Urticaria,” Journal of Allergy and Clinical Immunology 149, no. 6 (2022): 1819–1831. 35667749 10.1016/j.jaci.2022.04.010

[44] M. Maurer , P. Kolkhir , M. P. Pereira , et al., “Disease Modification in Chronic Spontaneous Urticaria,” Allergy 79, no. 9 (2024): 2396–2413. 39044706 10.1111/all.16243

[45] M. Maurer , W. Berger , A. Giménez‐Arnau , et al., “Remibrutinib, a Novel BTK Inhibitor, Demonstrates Promising Efficacy and Safety in Chronic Spontaneous Urticaria,” Journal of Allergy and Clinical Immunology 150, no. 6 (2022): 1498–1506. 36096203 10.1016/j.jaci.2022.08.027

[46] M. Maurer , T. B. Casale , S. S. Saini , et al., “Dupilumab in Patients With Chronic Spontaneous Urticaria (LIBERTY‐CSU CUPID): Two Randomized, Double‐Blind, Placebo‐Controlled, Phase 3 Trials,” Journal of Allergy and Clinical Immunology 154, no. 1 (2024): 184–194. 38431226 10.1016/j.jaci.2024.01.028

[47] M. Metz , A. Giménez‐Arnau , M. Hide , et al., “Remibrutinib in Chronic Spontaneous Urticaria,” New England Journal of Medicine 392, no. 10 (2025): 984–994. 40043237 10.1056/NEJMoa2408792

[48] Novartis NPC , Highlights of Prescribing Information for Rhapsido (Novartis NPC, 2025).

[49] M. Maurer , P. Staubach , U. Raap , G. Richter‐Huhn , M. Baier‐Ebert , and N. Chapman‐Rothe , “ATTENTUS, a German Online Survey of Patients With Chronic Urticaria Highlighting the Burden of Disease, Unmet Needs and Real‐Life Clinical Practice,” British Journal of Dermatology 174, no. 4 (2016): 892–894. 26406483 10.1111/bjd.14203

