# Supplementary material for: Unmet Needs in Treatment Escalation for Chronic Spontaneous Urticaria: Findings From the CURE Registry
Source: Allergy. 2026 Jan 14;81(8):2804–14. doi: 10.1111/all.70199 (PMC13466147; doi:10.1111/all.70199)
Supplement: Supplementary file 1 [file ALL-81-2804-s001.docx]

**SUPPLEMENTARY MATERIAL**

**Title**

Unmet needs in treatment escalation for chronic spontaneous urticaria: Findings from the CURE Registry

**Running title:** Real-world treatment patterns from CURE

**Authors**

Pavel Kolkhir,^1,2^ Pascale Salameh,^1–6^ Magdalena Zajac,^7^ Alicja Kasperska-Zajac,^7^ Ana Giménez-Arnau,^8^ Maria Puertolas,^8^ Hanna Bonnekoh,^1,2^ Carolina Vera Ayala,^1,2^  Michael Makris,^9^ Eleni Chatzidimitriou,^10^ Stamatios Gregoriou,^10^ Kanokvalai Kulthanan,^11^ Andrea Bauer,^12^ Mojca Bizjak-Suran,^13 –15^ Daria Fomina,^16–18^ Alexis Bocquet,^19^ Joachim Dissemond,^20^ Mohamed Abuzakouk,^21^ Tara Raftery,^22^ Nadine Chapman-Rothe,^23^ Emek Kocatürk,^1,2,24^ Clive Grattan,^25^ Riccardo Asero,^26^ Jonny G. Peter,^27,28^ Simon Francis Thomsen,^29,30^ Karsten Weller^1,2^

**Affiliations**

^1^Institute of Allergology, Charité – Universitätsmedizin Berlin, Corporate Member of Freie Universität Berlin and Humboldt-Universität zu Berlin, Berlin, Germany

^2^Fraunhofer Institute for Translational Medicine and Pharmacology ITMP, Immunology and Allergology, Berlin, Germany

^3^School of Medicine, Lebanese American University, Byblos, Lebanon

^4^Institut National de Santé Publique d'Épidémiologie Clinique et de Toxicologie-Liban (INSPECT-LB), Beirut, Lebanon

^5^Department of Primary Care and Population Health, University of Nicosia Medical School, 2417, Nicosia, Cyprus

^6^Faculty of Pharmacy, Lebanese University, Hadat, Lebanon

^7^European Center for Diagnosis and Treatment of Urticaria, Medical University of Silesia, Katowice, Poland

^8^Department of Dermatology, Hospital del Mar Research Institute, Universitat Pompeu Fabra, Barcelona, Spain

^9^Allergy Unit “D. Kalogeromitros”, 2nd Department, Dermatology and Venereology, National and Kapodistrian University of Athens, “Attikon” University Hospital, Athens, Greece

^10^National and Kapodistrian University of Athens, 1st department of Dermatology-Venereology, Andreas Sygros Hospital, Athens, Greece

^11^Department of Dermatology, Faculty of Medicine Siriraj Hospital, Mahidol University, Bangkok, Thailand

^12^Department of Dermatology, University Hospital Carl Gustav Carus, Technical University Dresden, Dresden, Germany

^13^Division of Allergy, University Clinic of Respiratory and Allergic Diseases Golnik, Golnik, Slovenia

^14^Faculty of Medicine, University of Ljubljana, Ljubljana, Slovenia

^15^ Faculty of Maribor, University of Maribor, Maribor, Slovenia

^16^Moscow City Research and Practical Center of Allergy and Immunology, State Budgetary Healthcare Institution, “Moscow Clinical Science and Research Hospital 52

of Moscow Healthcare Department”, Moscow, Russian Federation

^17^Department of Clinical Immunology and Allergology, Sechenov First Moscow State Medical University, Moscow, Russian Federation

^18^Department of Pulmonology, Astana Medical University, Astana, Kazakhstan

^19^Department of Internal Medicine and Clinical Immunology, CHU Grenobles Alpes, Grenoble, France

^20^Department of Dermatology, Venerology and Allergology, University of Essen, Essen, Germany

^21^Allergy & Immunology Department, Cleveland Clinic Abu Dhabi, Abu Dhabi, UAE

^22^Novartis Ireland Ltd., Dublin, Ireland

^23^Novartis Pharma AG, Basel, Switzerland

^24^Bahçeşehir University School of Medicine, Department of Dermatology, Istanbul, Turkey

^25^Guy’s Hospital, St John’s Institute of Dermatology, London, UK

^26^Ambulatorio di Allergologia, Clinica San Carlo, Paderno Dugnano, Milan, Italy

^27^Allergy and Immunology Unit, UCT Lung Institute, Cape Town, South Africa

^28^Division of Allergy and Clinical Immunology, Groote Schuur Hospital, University of Cape Town (UCT), Cape Town, South Africa

^29^Department of Dermatology, Bispebjerg Hospital, Copenhagen, Denmark

^30^Department of Biomedical Sciences, University of Copenhagen, Copenhagen, Denmark

**Corresponding authors:**

PD Dr. med. Karsten Weller, Institute of Allergology, Charité – Universitätsmedizin Berlin, Hindenburgdamm 30, 12203 Berlin, Berlin, Germany

Email: [karsten.weller@charite.de](mailto:karsten.weller@charite.de)

PD Dr. Pavel Kolkhir, Institute of Allergology, Charité – Universitätsmedizin Berlin, Hindenburgdamm 30, 12203 Berlin, Berlin, Germany

Email: pavel.kolkhir@charite.de

**Supplementary Table S1. Participating CURE centers contributing to this analysis.**

| **Country** | **City** | **CURE Center** |
| --- | --- | --- |
| Russia | Moscow | Clinical State Hospital 52 |
|  | Smolensk | Smolensk State Medical University |
|  | Moscow | NRC Institute of Immunology FMBA of Russia |
|  | Kazan | City Clinical Hospital No. 7 named after M.N. Sadykov |
| Germany | Gießen | Justus-Liebig-Universität Gießen |
|  | Berlin | Institute of Allergology, Charité – Universitätsmedizin Berlin |
|  | Buxtehude | Elbe Kliniken Buxtehude |
|  | Dresden | Uniklinikum Dresden |
|  | Essen | University Hospital Essen |
|  | Jena | Universitätsklinikum Jena |
|  | Mainz | University Medical Center of Johannes Gutenberg-University Mainz |
| Poland | Lublin | Independent Public Clinical Hospital No. 1 |
|  | Poznan | Poznan University of Medical Sciences |
|  | Zabrze | Europ. Center for Diagnosis and Treatment of Urticaria |
| France | Grenoble | Grenoble Alpes University Hospital |
|  | Paris | Assistance Publique-Hopitaux de Paris |
|  | Metz | Hospital Metz-Thionville |
|  |  |  |
|  | Montpellier | University Hospital of Montpellier Saint-Eloi |
| Spain | Barcelona | Hospital del Mar |
| Iran | Mashhad | Mashhad University of Medical Sciences |
|  | Teheran | Tehran University of Medical Sciences |
| Thailand | Bangkok | Mahidol University (Bangkok) |
| Greece | Athens | University General Hospital Attikon |
|  | Athens | National and Kapodistrian University of Athens |
| South Africa | Cape Town | University of Cape Town Lung Institute |
| Turkey | Istanbul | Istanbul Universıty, Department of Dermatology and Venereology |
|  | Kayseri | Kayseri City Education and Research Hospital |
|  | Istanbul | Koc University School of Medicine |
|  | Istanbul | Marmara University School of Medicine |
|  | Sakarya | Sakarya University Training and Research Hospita |
| Slovenia | Golnik | University Clinic of Respiratory and Allergic Diseases Golnik |
| United Arab Emirates | Abu Dhabi | Cleveland Clinic Abu Dhabi |
| Argentina | Bahia Blanca | IAIS- Instituto de Alergia e Inmunología del Sur |
|  | Buenos Aires | Hospital Italiano de Buenos Aires Argentina |
| Japan | Hiroshima | Hiroshima University Hospital |
|  | Kobe | Kobe University Graduate School of Medicine |
|  | Yokohama | Yokohama City University Hospital |
| China | Guangzhou | Sun Yat-sen Memorial Hospital |
|  | Suzhou | Second Affiliated Hospital of Soochow University |
| Italy | Milan | Clinica San Carlo Via Ospedale |
| Brazil | Salvador | Federal University of Bahia Medical School |
|  | São Paulo | Alergia e Imunologia Clinica |
| Netherlands | Rotterdam | Erasmus University Medical Center Rotterdam |
| Colombia | Medellín | IPS Universitaria |
| Portugal | Coimbra | University Coimbra_1 |
|  | Coimbra | University Coimbra_2 |
|  | Porto | Centro Hospitalar de São João |
| India | Bangalore | Kempegowda Institute of Medical Sciences (KIMS) and Research |
|  | Navi Mumbai | Dr.D.Y.Patil University and School of Medicine |
| Ireland | Dublin | St. James's Hospital |
| Bulgaria | Sofia | Medical Centre "Doverie SBT" |
| Denmark | Copenhagen | Bispebjerg Hospital |
| Mexico | Mexico City | Hospital Español de México |
| Macedonia | Skopje | University Skopje |
| Saudi Arabia | Hafar Al-Batin | King Khalid General Hospital |
| Ecuador | Guayaquil | RespiraLab Research Group |
| Croatia | Zagreb | Specijalna bolnica za plucne bolesti |

Co-authors were invited/included according to the CURE charter, i.e., based on the number of patient entries available for analysis from each center according to the version of the CURE charter on the date of the data cut. Centers that did not qualify for co-authorship are acknowledged in the table.

**Supplementary Table S2.** Sensitivity analysis based on a comparison of baseline age, disease duration, disease activity scores, and use of sgH_1_-AH between patients who had data at follow-up versus those who did not have these data.

|  | **Data available  at FU** | **Data not available  at FU** | **p-value**^†^ |
| --- | --- | --- | --- |
| **Age**, years (SD); N | 46.81 (15.25);1288 | 44.68 (15.42); 2707 | <0.001 |
| **Disease duration**, months (SD) | 14.68 (53.72); 1009 | 28.78 (350.24); 2184 | 0.067 |
| **Patients using sgH_1_-AH**,  % (n/N)  Licensed dose  Up dosed | 25.8% (332/1288)  29.5% (380/1288) | 35.7% (967/2707)  26.1% (707/2707) | <0.001  0.025 |
| **UCT BL,** score (SD); N | 7.77 (4.82); 992 | 8.50 (4.53); 2015 | <0.001 |
| **UAS7 BL,** score (SD); N | 19.20 (13.72); 801 | 15.90 (13.48); 1190 | <0.001 |
| **DLQI BL,** score (SD); N | 8.80 (7.20); 632 | 7.59 (6.61); 120 | <0.001 |
| **CU-Q_2_oL BL,** score (SD); N | 32.73 (21.05); 542 | 30.14 (19.25); 932 | 0.019 |

^†^two-sided.

BL, baseline; CU-Q_2_oL, Chronic Urticaria Quality of Life Questionnaire; FU, 6-month follow-up; DLQI, Dermatology Life Quality Index; n, number of patients receiving sgH_1_-AH; N, total number of patients; SD, standard deviation; sgH_1_-AH, second-generation H_1_-antihistamines; UAS7, weekly Urticaria Activity Score; UCT, Urticaria Control Test.

**Supplementary Table S3.** Treatment patterns based on UCT score at baseline and follow-up.

| **Therapy** | **Step-down at follow-up** | | | **No change at follow-up** | | | **Step-up at follow-up** | | | **Total, n (n with UCT available at FU)** |
| --- | --- | --- | --- | --- | --- | --- | --- | --- | --- | --- |
|  | *Total n with UCT<12 at BL, n (n with UCT available at FU)* | *UCT<12 at FU, %, n/N* | *UCT≥12 at FU, %, n/N* | *Total n with UCT<12 at BL, n (n with UCT available at FU)* | *UCT<12 at FU, %, n/N* | *UCT≥12 at FU, %, n/N* | *Total n with UCT<12 at BL, n (n with UCT available at FU)* | *UCT<12 at FU, %, n/N* | *UCT≥12 at FU, %, n/N* |  |
| No treatment | - | - | - | 27 (15) | 46.7, 7/15 | 53.3, 8/15 | 35 (28) | 60.7, 17/28 | 39.3, 11/28 | 62 (43) |
| sgH_1_-AH licensed dose | 44 (21) | 42.8, 9/21 | 57.1, 12/21 | 62 (55) | 32.7, 18/55 | 67.3, 37/55 | 47 (44) | 45.4, 20/44 | 54.6, 24/44 | 153 (120) |
| sgH_1_-AH  up-dosed | 83 (45) | 55.5, 25/45 | 44.4, 20/45 | 77 (73) | 45.2, 33/73 | 54.8, 40/73 | 47 (41) | 41.5, 17/41 | 58.5, 24/41 | 207 (159) |
| Omalizumab licensed dose | 14 (8) | 75.0,  6/8 | 25.0,  2/8 | 31 (25) | 32.0,  8/25 | 68.0,  17/25 | 1 (1) | 0.0,  0/1 | 100.0,  1/1 | 46 (34) |

BL, baseline; FU, follow-up; sgH_1_-AH, second-generation H_1_-antihistamines; UCT, Urticaria Control Test

**Figure S1. Data analysis flow chart.** Analysis performed on data from patients receiving no treatment, licensed dose sgH_1_-AH, up-dosed sgH_1_-AH (up to 4 times licensed dose) and omalizumab (licensed dose) with UCT score available at baseline and follow-up.

n, number of patients included in the analysis; N, total number of patients; sgH_1_-AH, second generation H1-antihistamines; UCT, Urticaria Control Test.

As shown in Table 2, a proportion of patients were on high-dose omalizumab (at BL=1.1%; at FU=1.8%), ciclosporin (at BL=0.6%; at FU=1.4%), and other treatments (at BL=13.7%; at FU=13.7%), had UCT score available, and were outside the focus of the main analysis.
